# Primigravida with Hypovolemic Shock Secondary to Ruptured Rudimentary Horn Ectopic Pregnancy: A Case Report

**DOI:** 10.31729/jnma.8617

**Published:** 2024-06-30

**Authors:** Siddharath Yadav, Indra Yadav, Sabita Jyoti, Prasanna Lama, Rozy Yadav

**Affiliations:** 1 Department of Obstetrics and Gynaecology, Birat Medical College Teaching Hospital, Biratnagar, Nepal; 2 Department of Community Medicine, Nepalgunj Medical College Teaching Hospital, Banke, Nepal; 3 Department of Community Medicine, Kathmandu Medical College, Sinamangal Kathmandu, Nepal; 4 Department of Obstetrics and Gynaecology, Province Hospital, Birendranagar, Surkhet, Nepal

**Keywords:** *case reports*, *ectopic pregnancy*, *hypovolemic shock*, *primigravida*, *rudimentary horn*

## Abstract

Developmental anomalies of genital tract result from defective fusion and absorption of various parts of Mullerian ducts in fetal life. Rudimentary horn pregnancy is a rare occurrence of one in 76,000150,000. We present a case of a 24-year-old primigravida with ruptured rudimentary horn pregnancy initially managed in the line of an intrauterine pregnancy with severe anemia. Hemodynamic instability made us suspect ruptured rudimentary horn pregnancy and lifesaving laparotomy was performed for the same. A 1.5 liter hemoperitoneum was encountered with a right rudimentary horn pregnancy. Multiple adhesions were present with necrotic tissue adherent and clumped together as tubo-ovarian mass. Resection of rudimentary horn was performed. We report this case to emphasize the need to consider rare uterine anomalies as a possibility in patients presenting with acute abdomen in early pregnancy. So, obstetricians can consider these rare entities in differential diagnosis and management.

## INTRODUCTION

The incidence of rudimentary horn pregnancy(RHP) is one in 76,000-150,000 pregnancies.^1^ About 8% of RHP are diagnosed before any symptoms appear.^2^ Mauriceau and Vassal in 1669 described rudimentary horn in pregnancy and in 1950, Latto et al described first case of ruptured RHP treated by laparotomy.^13^ The prevalence of unicornuate uterus is 0.1 % in general population.^4^ It results due to abnormal development of Mullerian ducts during embryogenesis.^4,5^ RHPs are rare however, associated with raised maternal morbidity and mortality.^5^

We report a case of a pregnancy, in a noncommunicating rudimentary horn of a unicornuate uterus without specific symptoms, incidentally found during operation and treated through laparotomy.

## CASE REPORT

A 25 years old primi gravida at 16 weeks and 5 days of gestation presented to the Emergency Department of a Teaching Hospital with a history of pain in the abdomen for 17 hours. The pain was associated with multiple episodes of vomiting. She confirmed her pregnancy through a urine pregnancy testing (UPT) kit at home four months back and had no antenatal care (ANC) thereafter. There was no history of altered bowel habits. There was no history of any chronic diseases like tuberculosis, diabetes, and hypertension.

On physical examination, her general condition was ill looking and Glass coma scale (GCS) was 15/15. Her blood pressure was 80/40 mm Hg, pulse was 160 beats per minute, respiratory rate was 26 breath per minute, temperature was 97 degree Fahrenheit and saturation of peripheral oxygen (SpO_2_) was 98 percent at room air. She looked pale and no abnormality was detected on her chest examination. On abdominal examination, the abdomen was distended, flank was full, tense and tender, and guarding was present all over the abdomen.

Per vaginal examination revealed closed os and positive cervical motion tenderness (CMT). She was resuscitated with intravenous fluid and noradrenaline. Abdominal ultrasonography (USG) showed an empty uterus, single fetus with no cardiac activity corresponding to 16 weeks and 5 days of gestation and placenta was seen in the abdomen. Cavity free fluid was present in Cul de sac and morrison pouch. Liver and kidney were grossly normal. USG diagnosed Primi ectopic pregnancy at 16 weeks and 5 days of gestation. Patient was prepared for an emergency laparotomy. Two pin packed red blood cells (PRBC) were arranged as the patient's haemoglobin was only 6 gram percent with blood group 'A' positive.

**Figure 1:**
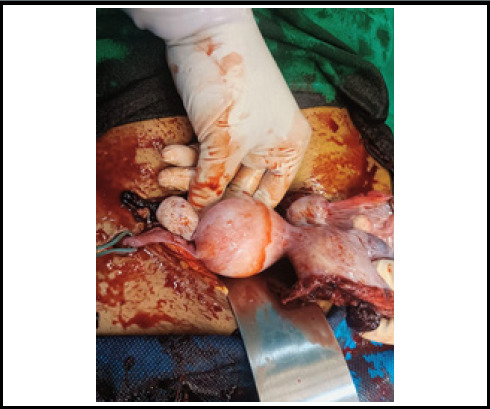
Rupture anterior wall of left noncommunicating rudimentary horn.

**Figure 2:**
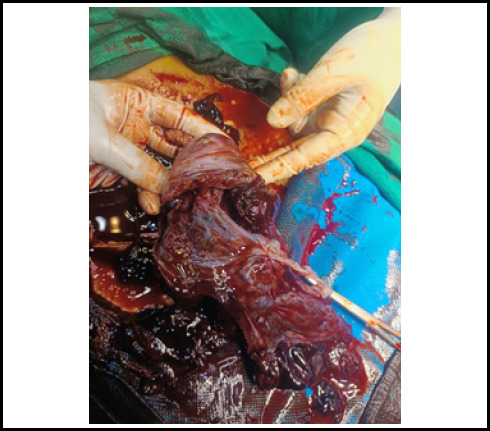
Placenta partially attached to rudimentary horn.

**Figure 3:**
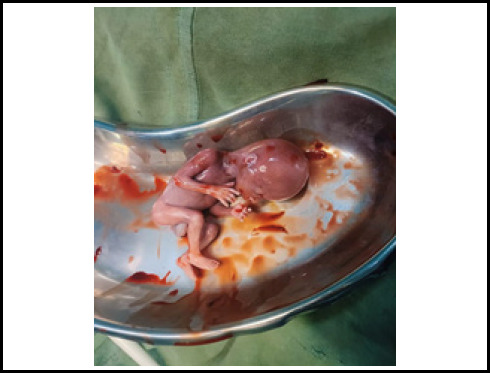
Dead female fetus.

**Figure 4:**
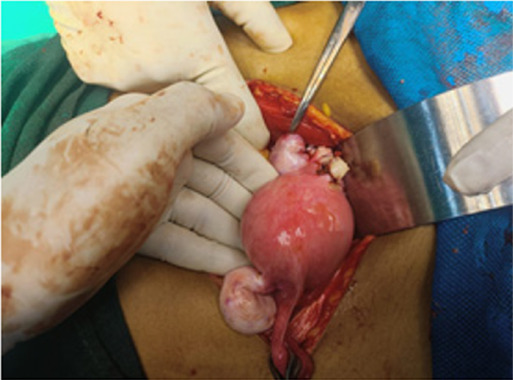
Resection of rudimentary horn and repair.

During the operation hemoperitoneum of 3500 ml was found. On exploring, diagnosis of primigravida with ectopic pregnancy at 16 weeks and 5 days of gestation with hypovolemic shock second degree to rupture abdomen and rudimentary horn pregnancy was made. There was a rupture present in the anterior wall of the left non-communicating rudimentary horn (Figure 1). Dead female fetus 300 grams with placenta Q1s removed from pT2toneum cavity and partially attached to rudimentary horn (Figure 2 and 3). However, a unicornuate uterus with normal ovary and tubes was seen. The rudimentary horn was attached to the unicornuate uterus with fibrous band. Round ligament and ovary were directly attached with the left rudimentary horn and was clamped. The resection of rudimentary horn was performed and ligation was done (Figure 4). Intraoperatively II pint of PRBC transfusion was done.

## DISCUSSION

We report a case of a non-communicating rudimentary horn of unicornuate uterus without specific symptoms because of a rare mullerian duct anomaly incidentally found during operation and treated through laparotomy. The patient was unaware as she did not have even single ANC and also the radiologist even could not detect the case when she presented to our hospital setting. These type of anomaly is difficulty to diagnosis as there is no clear clinical criteria, and mostly diagnosis when it's already rupture. The ultrasonography sensitivity is also limit as only 26% and decreased with advancing gestational age. This is the reason few cases are only reported in the first trimester. However, early diagnosis to prevent complications of such life threatening conditions is essential.^1,6,7^

Depending upon the horn musculature and also its ability to hypertrophy, the time of uterine rupture varies from 5 to 35 weeks. The thickness and vascularity of the uterine walls and gestational age are directly proportional with increase and severe blood loss.

So, such cases can be prevented through laparoscopic route examination through excision of rudimentary horn but it requires diagnosis in the first trimester which is very challenging. Another alternative therapeutic producer for treatment in first trimester is injection methotrexate into chronic cavity or its systemic use later excision of rudimentary horn. While comparing success rate methotrexate injection local under laparoscopic or ultrasonographic guidance was 91%, its systemic use was 79%.^1,5,7,8^

This case report concludes that RHP is a rare condition that may be misdiagnosed as normal pregnancy during ANC, where USG may also not be conclusive before surgery. However, it is associated with high maternal morbidity and mortality. We encourage more supervision and new guidelines and diagnostic equipment and radiological support for early diagnosis in near future.
